# Micro-gun based on laser pulse propulsion

**DOI:** 10.1038/s41598-017-16400-7

**Published:** 2017-11-24

**Authors:** Haichao Yu, Hanyang Li, Lugui Cui, Shuangqiang Liu, Jun Yang

**Affiliations:** 1 Key Lab of In-fiber Integrated Optics, Ministry Education of China, Harbin Engineering University, Harbin, 150080 China; 2 Department of Physics, Harbin Engineering University, Harbin, 150080 China

## Abstract

This paper proposes a novel “micro-gun” structure for laser pulse propulsion. The “micro-bullets” (glass microspheres) are irradiated by a laser pulse with a 10 ns duration in a dynamic process. Experimental parameters such as the microsphere diameter and the laser pulse energy are varied to investigate their influence on laser pulse propulsion. The energy field and spatial intensity distribution in the capillary tube were simulated using a three-dimensional finite-difference time-domain method. The experimental results demonstrate that the propulsion efficiency is dependent on the laser pulse energy and the microsphere size. The propulsion modes and sources of the propelling force were confirmed through direct observation and theoretical calculation. Waves also generated by light-pressure and thermal expansions assisted the propulsion.

## Introduction

Laser pulse propulsion (LPP) has attracted large prominence due to its non-contact nature and ability to affect the motion of objects ranging from various macroscopic materials^1–3^ to microscopic objects and even individual microspheres^4,5^. LPP has the potential to increase the payload and decrease the launch costs in comparison with other conventional methods of producing thrust^6–8^. Researchers have spent decades exploring the governing interaction mechanism of LPP through experimental phenomena, especially the micro aspects, such as the propulsion of micro-scale microspheres made of various materials^9^. A microsphere is surrounded by air molecules; the atoms or molecules in the air absorb the energy of multiple photons after the microsphere is irradiated by a single laser pulse. Photoionization can occur when the energy of the photons is higher than the ionization energy of the atoms or molecules. The electrons escape from the atoms or molecules and become free electrons, which absorb laser pulse energy via inverse bremsstrahlung. High-density and high-temperature plasma is formed once the free electrons reach a certain density (10^18^/cm^3^)^10,11^. The motion of objects results from the impact of the plasma at the surface of the materials.

Additionally, several other mechanisms may be involved in this process, such as thermoelastic waves^12^ generated by energy absorption and the thermal expansion of the substance. These effects may permanently damage the surface structures or even create fragments^13^. A transmission method (e.g., coupling the laser pulse with an optical fibre) can be used to protect the microsphere from laser-induced surface damage^14^, but may offset the microsphere’s trajectory due to the spatial distribution difference of the plasma density^15^. The difficulties that are associated with experiments create limitations concerning the analysis of LPP, and most previous studies have focused on LPP rather than the propulsion modes or dynamic sources.

LPP not only can be utilized for advanced studies in military science, aerospace, and propulsion efficiency fields^16^, such as techniques for the removal of particles from target surfaces^17^, but also has potential applications in microinjection and drug delivery^18^. According to the different requirements, particles with various characteristics can be fabricated and can be propelled by light. For example, in medical applications, by using light to transfer energy to microparticles, the microparticles can be propelled in the blood vessel by adjusting the direction of the light source. LPP as a potential propulsion technology can be used for punctual delivery of drugs to body tissues and can thus reduce the cell death rate. With detailed study of the characteristics of particles, laser propulsion of micro-sized particles could be achieved, and the drug delivery could be realized.

Here, we address these limitations by developing a novel “micro-gun” structure. The capillary tube serves as a guiding tool to limit the movement of the microspheres and reduce the loss of laser pulse energy. The propulsion modes and source origins were demonstrated in our experiment. Furthermore, a smaller, micro-scale “micro-gun” based on our setup may have a significant influence on nanofluidic injection and drug delivery.

## Results

### The “micro-bullet” dynamic processes analysis

**Figure 1:**
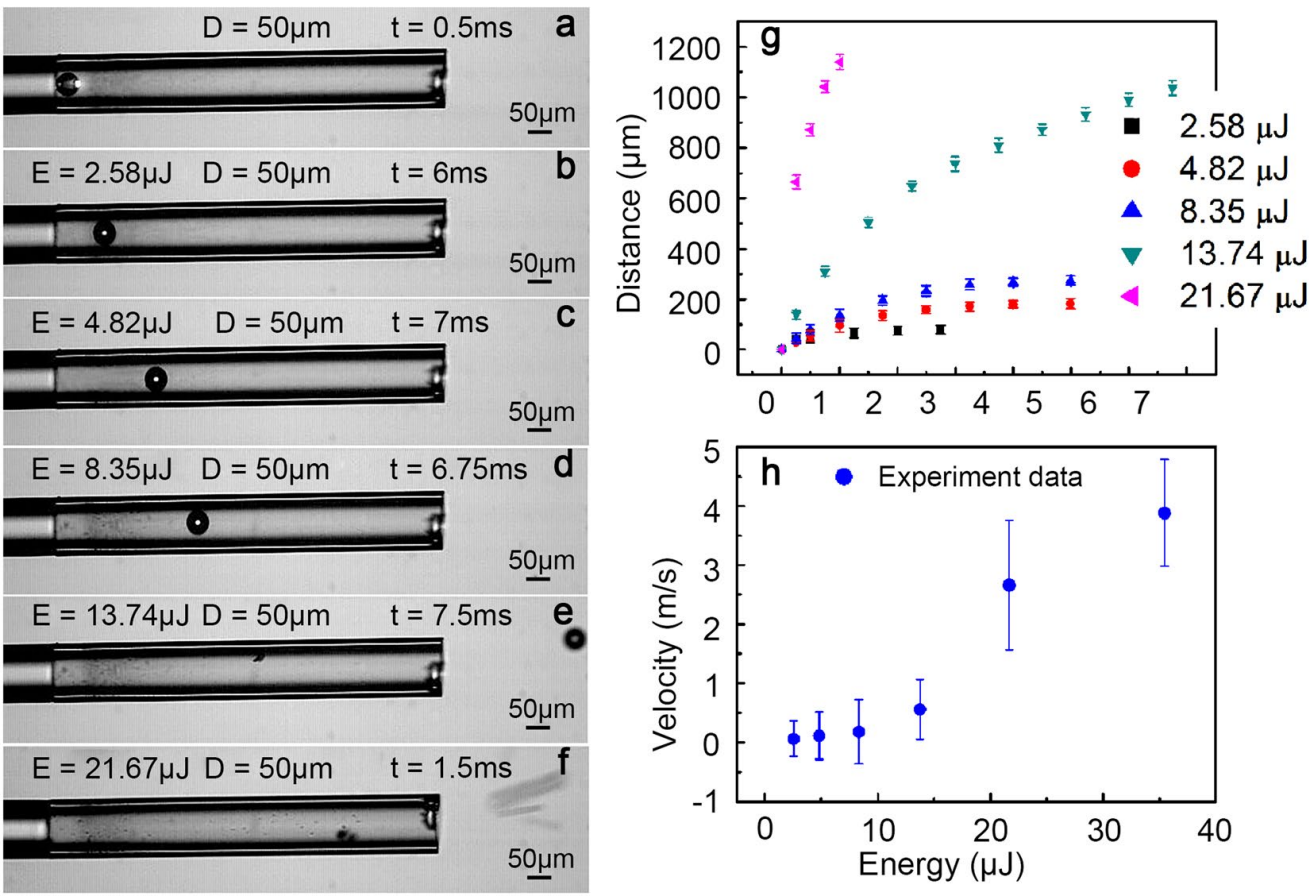
The moving trace of a microsphere with a diameter of 50 μm at various laser pulse energies. (**a**) Initial state. (**b**) 2.58 μJ. (**c**) 4.82 μJ. (**d**) 8.35 μJ. (**e**) 13.74 μJ. (**f**) 21.67 μJ. (**g**) Variation of the microsphere moving distance with time. (**h**) The initial velocity of microsphere’s dependence on the laser pulse energy launched from the fibre. See Supplementary video 1.

Plasma was generated when the microsphere was irradiated by the laser pulse, and then the microsphere was moved due to plasma expansion^19^. The glass microsphere (50 μm) dynamic processes at laser pulse energies of 2.58, 4.82, 8.35, 13.74, and 21.67 μJ are shown in Fig. 1a–f. The first frame of the microsphere attachment fibre section in the capillary tube is marked as *t* = 0.5 ms (Fig. 1a), where an approximately elliptical plasma plume appears (see Supplementary Fig. S1) since the plasma in the incident laser pulse direction has greater kinetic energy than that in the direction perpendicular to the laser pulse^20,21^. As expected, the laser pulse energy increased the movement distance as the initial velocity increased, as reported in Table 1. At the laser pulse energy of 13.74 μJ, the initial momentum of the microsphere was 9.5 × 10^−11^ N∙s (Fig. 1e), and the microsphere was damaged (see Supplementary Fig. S2).

**Table 1:** Movement distance and initial velocity of the microsphere with a diameter of 50 μm.

Energy (μJ)	2.58	4.82	8.35	13.74	21.67
Movement distance (μm)	80.4	183.8	275.2	1,036	1,140
Initial velocity (m/s)	0.076	0.115	0.182	0.556	2.6

As shown in Fig. 1d–f, smaller particles formed and attached to the inner wall of the capillary due to vapour condensation. We investigated the mechanisms by studying the interaction between the laser pulses and the microsphere. Based on *T*_m_ ≈ *T*_0_ + (*F*/*c*_i_) *α* ^22^, where *T*_0_ = 300 K is room temperature; *c*_i_ = 750 J/(kg⋅K) is the specific heat of glass microsphere; *α* ≈ 4.1 × 10^6^ is a comprehensive coefficient, which is the integrated computation values^23^ of the light attenuation coefficient, the thermal conductivity and the electron-phonon coupling factor; *F* = (1 − *R* − *τ*)*E* is the absorbed laser fluence, where *E* is the laser pulse energy and *τ* is the optical transmissivity. The calculation processes are shown in “Methods” section. We found that the maximum surface temperature (~735 K) exceeded the softening temperature (~700 K) at the energy of 8.35 μJ. Smaller particles were generated in the direction to opposite that of the incoming laser pulse and attached to the capillary tube. At laser pulse energy *E* < 8.35 μJ, the plasma expansion was the main driving force, and the corresponding propulsion mode was the air breathing mode. At the laser pulse energy of 8.35 μJ or above, the dynamic source was both plasma and vapour expansion, and propulsion occurred in both the air breathing mode and the ablation mode. The experimental data of LPP for glass microsphere at various laser pulse energies are shown in Fig. 1g and h, and the corresponding image sequences are shown in Fig. 1a–f. In Fig. 1h, the velocity of the microsphere is proportional to the laser pulse energy.

**Figure 2:**
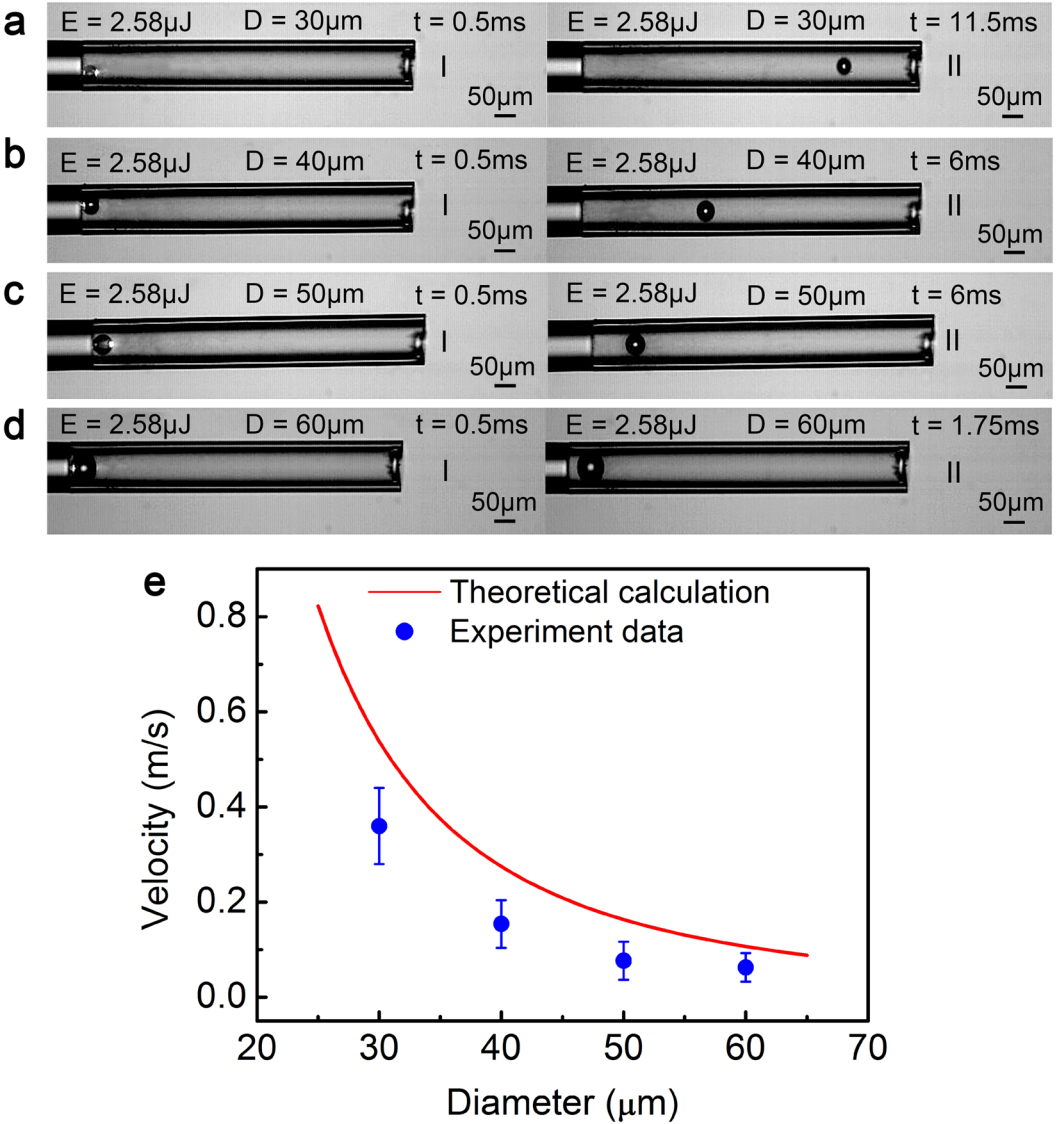
Microsphere trajectories at a constant laser pulse energy (2.58 μJ). (**a**–**d**) represent 30, 40, 50, and 60 μm, respectively. (**e**) Movement velocity of different sizes of microspheres with theoretical calculations. See Supplementary video 2.

Next, we investigated the influence of microsphere size on the LPP. Image sequences of microspheres (30~60 μm) at the same laser pulse energy (2.58 μJ) are shown in Fig. 2. The initial state, aI - dI, is marked at *t* = 0.5 ms, and aII~dII represents the final state. The movement distance and initial velocity of the microsphere are shown in Table 2. We found that the movement distance and velocity of microsphere appear to be non-proportional to the increase in the microsphere size^17^. The main dynamic source was plasma expansion, and the corresponding propulsion mode occurred in air breathing mode. The qualitative distributions of the experimental data agree with our theoretical calculations (Fig. 2e), although the experimental data are below the theoretical calculation curve.

**Table 2:** Movement distance and initial velocity of the microsphere at the energy of 2.58 μJ.

Diameter (μm)	30	40	50	60
Movement distance (μm)	604.8	276	80.4	19.92
Initial velocity (m/s)	0.365	0.154	0.076	0.063

### Comparison of the movement distances of microspheres at an energy of 4.82 μJ

**Figure 3:**
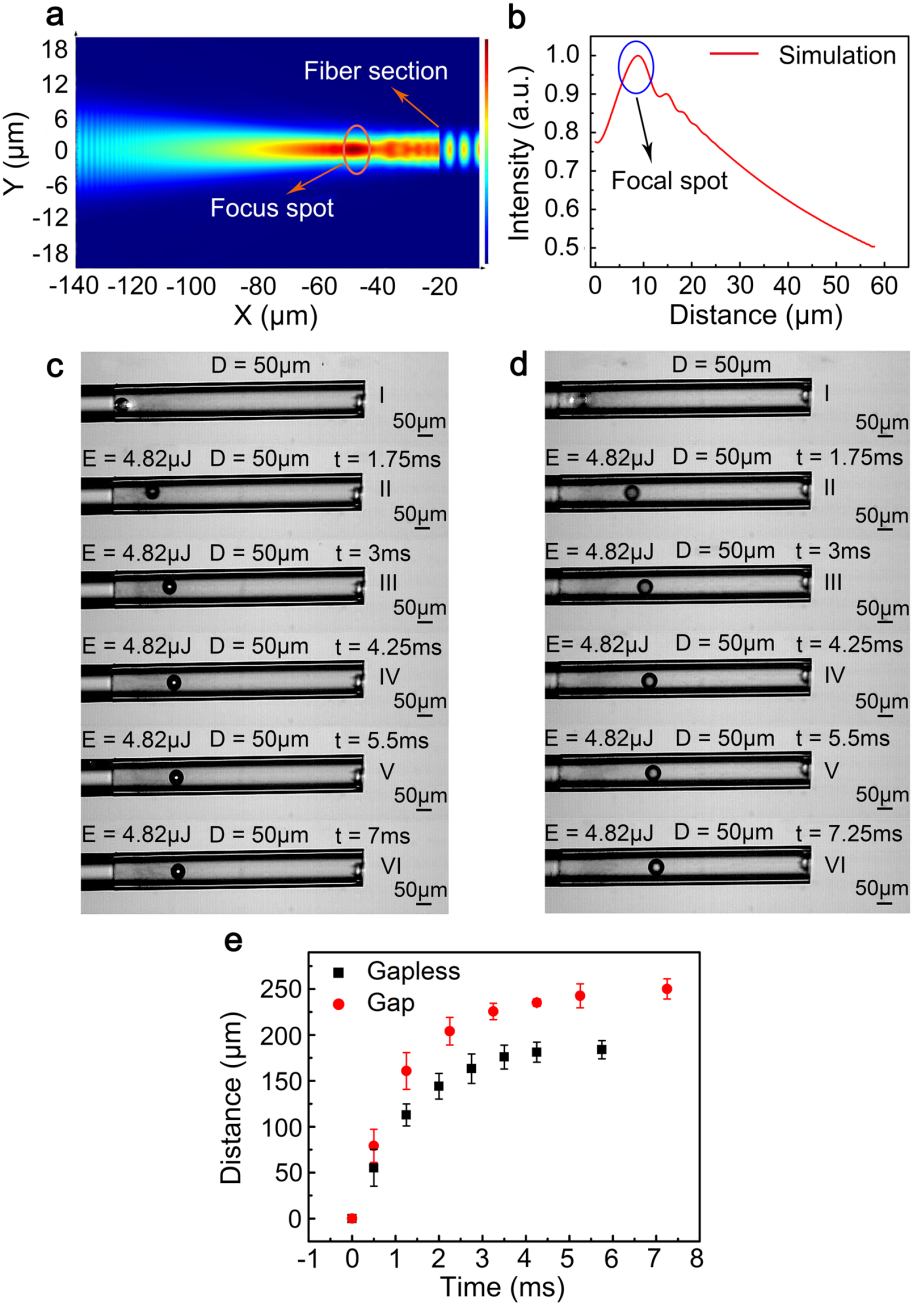
Comparison experiment at the energy of 4.82 μJ. (**a**) Fibre section energy field spatial distribution. (**b**) Normalized energy field intensity distribution. (**c**) Microsphere moving without a gap; recording times of I–VI: 0.5, 1.75, 3, 4.25, 5.5, and 7 ms. (**d**) Microsphere moving with a gap; recording times of I–VI: 0.5, 1.75, 3, 4.25, 5.5, and 7.25 ms. (**e**) Movement distance as a function of time. See Supplementary video 3.

The three-dimensional finite-difference time-domain model is employed to determine whether the microsphere’s location affects its motion. Figure 3a and b show side views of the energy field and the intensity spatial distribution, with a high energy intensity at a distance of approximately 30 μm (red region); i.e., the laser pulse was focused by a fibre. While the microsphere was located in the “focal spot”, the movement distance and the initial velocity increased.

In an additional experiment, one 50-μm-diameter microsphere was attached to a fibre section and another was placed on the “focal spot”. The initial velocities were 0.115 m/s for the gapless condition (Fig. 3c) and 0.134 m/s for the gap (Fig. 3d) condition. We can observe that the microsphere moved slightly further when a gap between the fibre section and the microsphere was present (Fig. 3e).

Simulation and experimental results together showed that the laser pulse in our setup is focused by the fibre. Thus, a larger reaction force acts on the surface when the microsphere is situated within the focal spot. In this manner, the propulsion efficiency can be improved significantly. A simplified analysis is provided to demonstrate that the propulsion efficiency is dependent on the microsphere size and the laser energy.

Generally, there are two important parameters for evaluating the efficiency of LPP. The momentum coupling coefficient (*C*_m_)^24^, which is defined as the ratio of the momentum to the laser pulse energy.

1 $${C}_{{\rm{m}}}=mv/E$$

where *m* and *v* are the mass and velocity of the microsphere and *E* is the energy of the pulse laser. It indicates the capacity for the laser pulse energy to be converted to kinetic energy.

**Figure 4:**
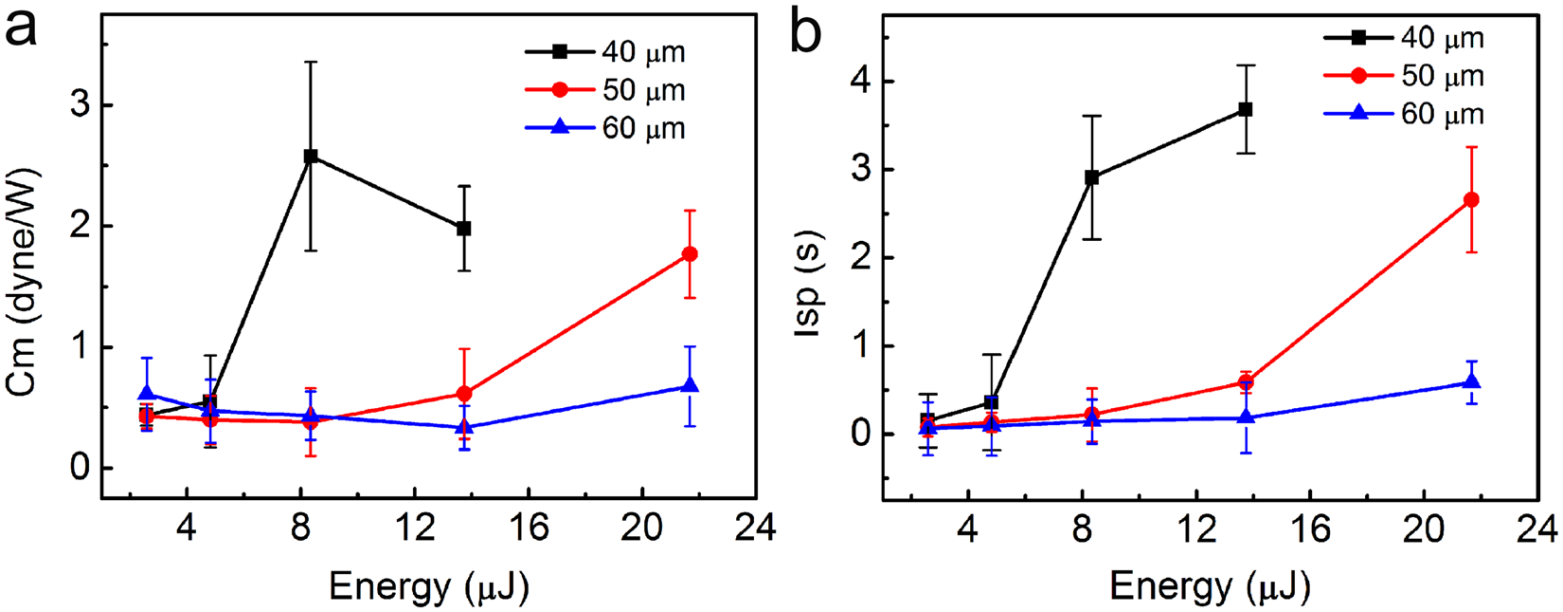
Propulsion parameters. (**a**) and (**b**) are the *C*_m_ and *I*_sp_ under different laser pulse energies for three sizes of microsphere, respectively.

Figure 4a shows the *C*_m_ generated from the LPP for three sizes of microspheres under various laser pulse energies. The optimum *C*_m_ value for the 40 μm microsphere is approximately 2.57 dyne/W at the laser pulse energy of 8.35 μJ, which decreases due to the laser plasma shielding effect. However, we cannot obtain the optimum *C*_m_ or optimum laser pulse energy for microspheres with a diameter of 50 or 60 μm. We assume this is because the optimum *C*_m_ value exceeded the region of the laser pulse energy, as shown in Fig. 4a.

The specific impulse (*I*_sp_)^25–27^ is the quotient between the target momentum and the consumption mass ratio:

2 $${I}_{{\rm{sp}}}=(mv)/({m}_{l}g)$$

where *m*_l_ is the consumption mass.

Figure 4b shows that the *I*_sp_ for the three sizes of microsphere is proportional to the increase in the laser pulse energy. We also cannot obtain the optimum *I*_sp_ for microspheres with diameters of 40, 50 and 60 μm.

## Discussion

We present LPP on dielectric microspheres using a novel “micro-gun” structure; the results that were obtained in the experiment helped develop a deeper understanding of the interaction mechanism and propulsion modes of nanosecond laser pulse propulsion of microspheres. LPP is an attractive technology, and Li *et al*.^17^ have demonstrated the feasibility of laser pulse propulsion on a glass microsphere. The energy fluence of the laser pulse outgoing from a tapered fibre exceeds the ionization threshold of air, which leads to plasma generation. The microsphere is driven by the plasma expansion. However, the moving trace of the microsphere deviates from the laser propagation direction in Li’s experiment. We have overcome this limitation by introducing a microstructure. Capillary tube serves as a guiding tool to limit the direction of the microsphere’s movement. Moreover, we have demonstrated the interaction mechanism, propulsion modes and propulsion efficiency in this paper. Another experiment that was conducted by Zheng *et al*.^28^. It demonstrated the effects of an Al target configuration on the propulsion efficiency. There is a cavity on the Al surface; the plasma expands is confined by the cavity, and the momentum coupling coefficient is improved by more than two orders of magnitude. In our experiment, a microstructure that acts as a confining geometric structure was proposed; it can reduce the loss of laser pulse energy and confine the plasma expansion, which leads to an enhanced propulsion efficiency.

We have demonstrated that the microsphere can be propelled when it is irradiated by a single laser pulse. A microstructure allows the guidance particles (e.g., microspheres and cells) along tailored trajectories. This technique might be applied in the field of drug delivery where the accelerated microparticles can be applied for localized treatments. Mark Jayson Villangca *et al*.^18^ have proposed a micro-tool for material transport that is capable of unloading and loading materials. The drug was unloaded using photothermally induced convection currents when the micro-tool reached body tissues, and this method has been successfully used in drug delivery^29^. This application was named targeted drug delivery, but the body tissue maybe damage while the drug process, it is necessary to further study. Another potential application of propulsion particles is cell feature detection in medical applications. The traditional measurement methods will average out the signature of this type of cell because the tumour cells are very rare in the blood. Microparticles can act as a probe to precisely realize the feature detection of a single tumor cell^30^.

**Figure 5:**
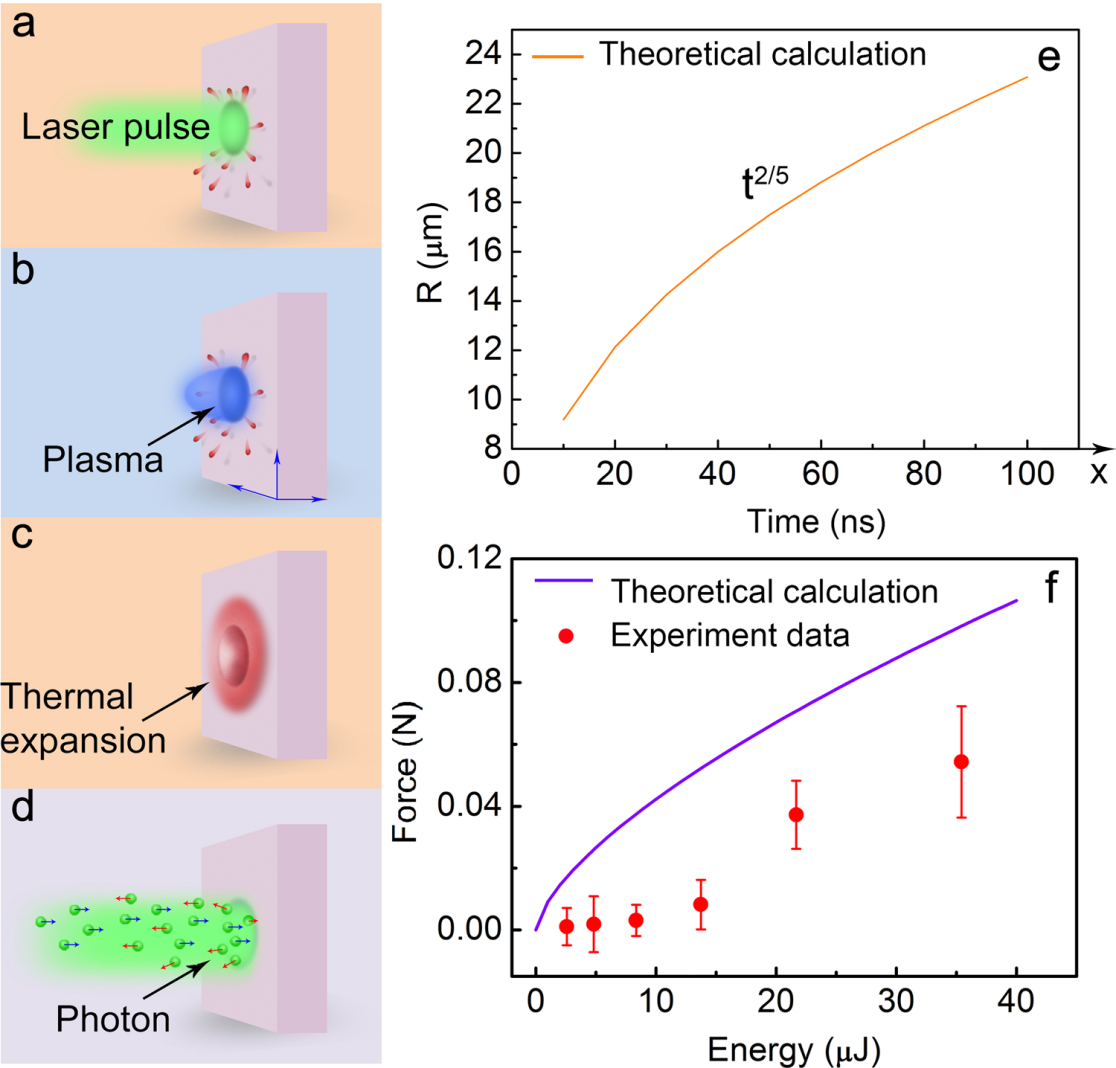
Conceptual scheme of various elastic waves and theoretical calculations. (**a**) Laser-matter interaction. (**b**) Plasma expansion. (**c**) Thermal expansion. (**d**) Light pressure. (**e**) Expansion distance of plasma in the radial direction. (**f**) Reaction force that is dependent on the laser pulse energy launched from the fibre, plotted with the theoretical calculation curve.

In the theoretical analysis, various types of waves are launched from the surface when the microsphere is impacted by a laser pulse (Fig. 5a–d). There are several sources of the reaction force^31^.

1) Plasma generation due to air ionization, with the corresponding propulsion mode is air breathing mode (Fig. 5b). The plasma radial expanding velocity is estimated to be several kilometres per second (Fig. 5e)^32^. The expansion distance as a function of time can be expressed as follows:

3 $$R=\lambda {(E/{\rho }_{0})}^{1/(2+\beta )}{t}^{2/(2+\beta )}$$

where *λ* ≈ 1 is a dimensionless constant, *ρ*_0_ = 1.3 kg/cm^3^ denotes the density of air, *t* is the propagation time and *β* is the spread type.

Here, we assume that the gas between the fibre section and the microsphere completely ionized when single laser pulse irradiation on the glass microsphere. The mass of air is *m*_A_ = *ρ*_0_ *V*_A_ = 1.3 × [π (25 × 10^−6^)^3^ − 0.5 × (4π/3) × (25 × 10^−6^)^3^] kg, and the mass of the microsphere is *m*_B_ = (4π/3) × (25 × 10^−6^)^3^ kg. Based on the momentum conservation law, *m*_A_ *v*_A_ = *m*_B_ *v*_B_, the expanding speeds in the laser pulse incoming direction are range from 0.38 to 25 km/s^33,34^.

2) The thermal vapour expansion associated with micro-structure formation at the surface of the microsphere (Fig. 5c).

3) Light pressure, where wave-particle duality causes momentum transfer when the surface of the microsphere is impacted by photons (Fig. 5d). It should be mentioned that plasma formation leads to a significant decrease in the reflectance, as a part of the laser pulse energy is absorbed by free electrons via inverse bremsstrahlung^35–37^. Furthermore, Fig. 5f shows another theoretical calculation with respect to the pressure exerted on the microsphere surface. We found that the pressure is proportional to the increase in the laser pulse energy.

In our experiment, the experimental data are below the theoretical calculation curve; There are several possible reasons for this: 1) a non-uniform force acting on the surface of the microsphere due to offset between the centre of the microsphere and the fibre; 2) the microsphere’s size left a narrow gap between the microsphere and the capillary tube, which allowed air flow in the capillary and affected the movement of the microsphere; 3) friction between the microsphere and the capillary tube.

Moreover, the capillary tubes can be seen as blood vessels, the microsphere can be seen as drug. Therefore, LPP may be a potential application for understanding the field of biology at the micro level.

## Conclusion

In summary, we present LPP on dielectric microspheres using a novel “micro-gun” structure that operates under normal indoor conditions. The microstructure can lead to an improvement in propulsion due to plasma expansion confinement. Laser pulse energy from the fibre section transforms into kinetic energy of a glass microsphere, and the motion process of the microsphere is demonstrated. Based on the law of momentum conservation, the movement velocity of the microsphere is calculated to be approximately 2.66 m/s. We also analysed the power origins at each test stage, laser energy *E* = 8.35 μJ as a demarcation point, the propulsion mode from the air breathing mode changes into the co-action of the air breathing mode and the ablative mode. The laser pulse was focused by the fibre, and the high intensity laser impulse on the surface while the microsphere was located in the “focal spot”. We found the laser-matter interaction mechanisms to be a function of the laser energy and the microsphere size. With further development, the LPP technique may have applications in the manipulation of biological matter and bacteria at the micro-scale level, such as in nanofluidic injection applications.

## Methods

### Experimental setup for LPP

**Figure 6:**
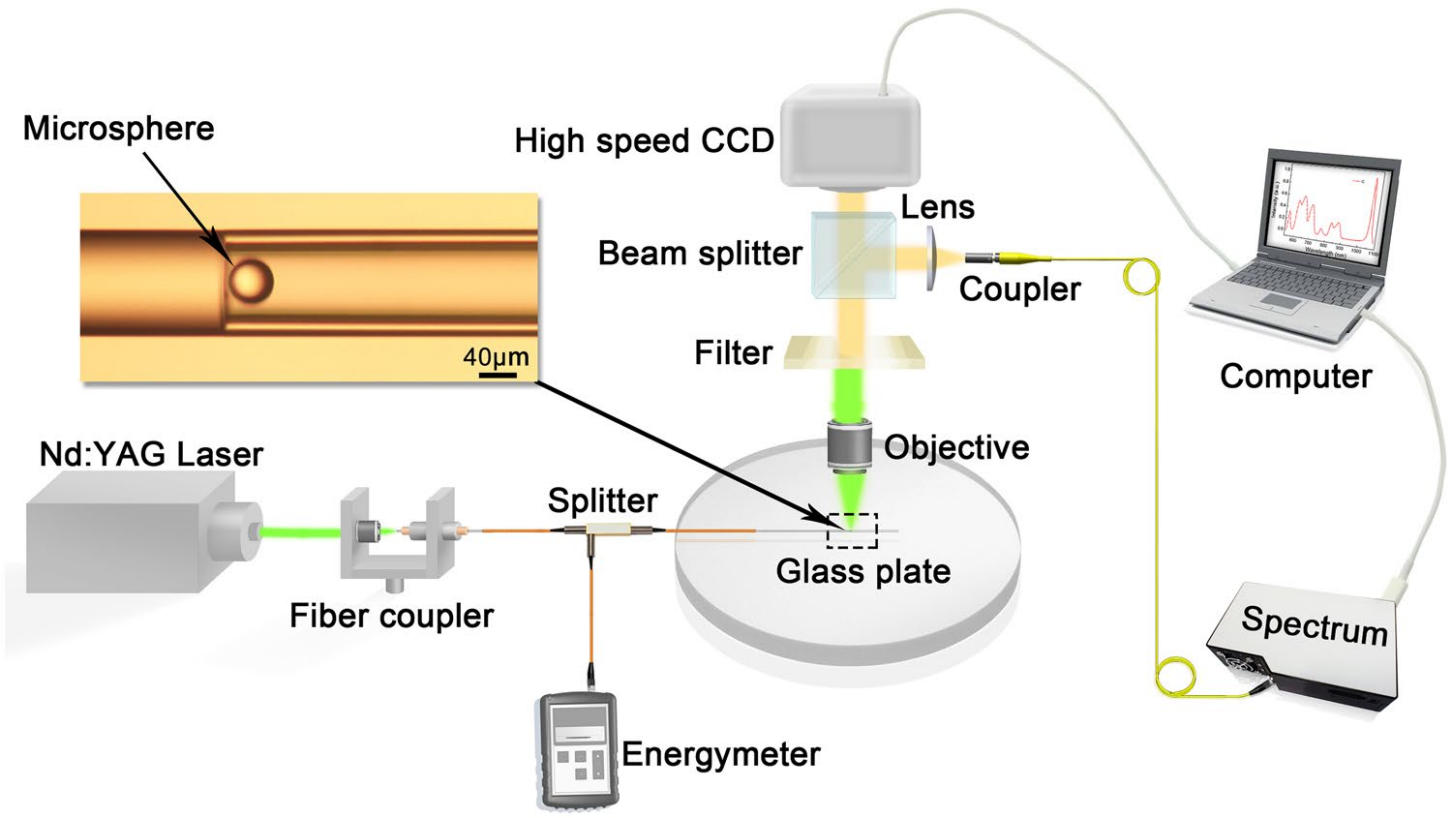
Schematic of the experimental setup for LPP.

Our experimental LPP setup is shown in Fig. 6. The “micro-gun” structure consists of a fibre and a capillary (TSP075150, Innosep Biosciences, internal and external diameter of 75 and 150 μm). We used a double frequency Nd: YAG laser (λ = 532 nm, pulse duration = 10 ns, 1–10 Hz) amplifier system. The pulses are coupled into a fibre by an objective (NA = 0.25). The pulse energy launched from the Nd: YAG was determined by adjusting the internal voltage control box of the laser amplifier system. Pulses accessed the energy meter (Field Maxll-Top, resolution is 0.01 μJ) and the “micro-gun” structure through a 50/50 coupler. Laser pulse energies from 2.58 to 21.67 μJ were measured. Microspheres (30~60 μm; the mass of the microsphere is proportional to *r* ^3^, where *r* is the radius of the microsphere) were pushed into a 75 μm diameter capillary tube by a tapered fibre (~30 μm) under a microscope, as shown in the upper left portion of Fig. 6. The transmission beam passing through the objective, the 532 nm filters and a beam splitter was collected by a high-speed CCD (Photron fastcam-ultima 1024, exposure time is 0.25 ms) camera with 4000 frames/s to record the dynamic processes. A spectrometer (Ocean Optics QE Pro, 1-nm resolution) was used for plasma spectrum acquisition. To decrease the experimental error, we repeated the experiments three times under identical experimental conditions.

### Plasma evolution processes

The laser pulse energy is absorbed by the microsphere, if the laser pulse energy fluence exceeds the threshold of air or microsphere ionization, the plasma is then formed, and the microsphere is accelerated. The plasma evolution process can be divided into three stages. First, air plasma is formed around the surface of the microsphere. The plasma then continuously expands in an approximately circular manner as it absorbs the laser pulse energy. Finally, the plasma expands freely and forms an elliptical plasma plume as more laser pulses are deposited in the incoming laser pulse direction^33^.

### Theoretical calculation of laser pulse energy absorbed by microsphere

The microsphere is irradiated by a single nanosecond laser pulse with the energy that is transmitted, absorbed, and reflected by the microsphere surface. The theoretical calculation formula of the laser pulse energy that is absorbed by a glass microsphere based on Bouguer’s law and refraction law is^38^:

4 $$\tau =\frac{1-R}{1+(2m-1)R}\cdot \exp (-kl/\,\cos \,\theta )$$

where *m* denotes the number of plies, *θ* denotes the refraction angle and *l* denotes the thickness of the glass microsphere.

For a visible laser pulse, reflectivity is expressed as follows:

5 $$R={[(1-n)/(1+n)]}^{2}$$

where *n* is the refractive index. Thus, the absorbance *F* can be written as:

6 $$F=1-R-\tau \approx 10 \% $$

### Theoretical calculation of the pressure and microsphere velocity

The expression of the theoretical calculation can be written as follows^39^:

7 $$P({\rm{Gpa}})=3.22\times {10}^{3}{(\frac{a}{2a+3})}^{2/3}{\rho }_{0}^{1/3}(g/{{\rm{cm}}}^{3})\times {I}^{2/3}({\rm{GW}}/{{\rm{cm}}}^{2})$$

where *α* = 0.4 denotes the energy coupling coefficient, *ρ*_0_ = 1.29 kg/m^3^ denotes the air density and *I* denotes the power density, which was determined by the laser pulse energy.

When a laser pulse strikes the microsphere, the absorption processes changes the pressure due to the plasma expansion that is transferred to the microsphere momentum based on the momentum conversation law. The relationship between the microsphere and the movement velocity is given by equation (8):

8 $$\frac{mv}{\pi {r}_{1}^{2}\tau }=3.22\times {10}^{3}{(\frac{a}{2a+3})}^{2/3}{\rho }_{0}^{1/3}(g/{{\rm{cm}}}^{3})\times {(\frac{2.58\times {10}^{-6}}{\tau \pi {r}_{2}^{2}})}^{2/3}$$

where *α* = 0.4 denotes the energy coupling coefficient, *v* denotes the velocity of microsphere, *τ* denotes the pulse duration, *ρ*_0_ = 1.29 kg/m^3^ denotes the density of air, *r*_1_ = 25 μm denotes the radius of microsphere and *r*_2_ = 25 μm denotes the radius of fibre core.

### The calculation of the microsphere’s initial velocity

The dynamic processes of the microspheres were recorded using a high-speed CCD camera with 4000 frame/s. *t* = 0 is declared as initial state, after 1/4000 s. The movement distance *S* of the microsphere can thus be determined from the recorded time series. To decrease the experimental error, we repeated the experiments three times under identical experimental conditions. Subsequently, the average velocity *v* = *S/t* of the microsphere was obtained. We consider the average velocity as the initial velocity due to the short interaction time between the laser pulse and the microsphere.

### Simulation of the energy field and intensity distribution

Simulation software named FDTD was employed to simulate and calculate the energy field and the intensity distribution. First, we built the simulated mode system, which includes the fibre and the substrate. We selected fused silica optical windows as the substrate. Considering the symmetric structure and the boundary conditions, we reduced the model to a 2D model. The schematic of the FDTD simulation configuration is shown in Supplementary Fig. S3. Then, we selected a 532 nm laser pulse as the light source. The light source propagated along the fibre. The diameter of the fibre is 125 μm, the refractive index are *n*_SiO2_ = 1.45 and *n*_Air_ = 1.

### The plasma formation mechanism

In our experiment, plasma expansion plays an important role as the main dynamic source, but the plasma formation regime involves a very complex process, and many processes are involved during the formation stage. We are considering that the plasma is formed through the regimes of multi-photon ionization (MPI) and avalanche ionization. In MPI, the energy of multiple photons is absorbed by the atoms or molecules. The photon energy is higher than the ionization energy of the atoms or molecules. The electrons escape from the atom and become free electrons:

9 $$M+\,\text{mh}{\rm{\nu }}\to {{\rm{M}}}^{+}+{{\rm{e}}}^{-}$$

In avalanche ionization, a number of free electrons sever as “seed electrons”. Subsequently, the “seed electrons” collide with atoms, and can each produce two free electrons with lower speeds.

10 $${{\rm{e}}}^{-}+{\rm{M}}\to {{\rm{M}}}^{+}+2{{\rm{e}}}^{-}$$

High-temperature and high-pressure plasma is formed once free electrons reach a certain density (10^18^/cm^3^).

## Electronic supplementary material

supplementary information

Supplementary Video 1

Supplementary Video 2

Supplementary Video 3

Supplementary Video 4
