# Supplementary material for: Micro-gun based on laser pulse propulsion
Source: Sci Rep. 2017 Nov 24;7:16299. doi: 10.1038/s41598-017-16400-7 (PMC5701249; doi:10.1038/s41598-017-16400-7)
Supplement: Supplementary file 1 — supplementary information [file 41598_2017_16400_MOESM1_ESM.pdf]

Supplementary Information

Micro-gun based on laser pulse propulsion

Haichao Yu[1,+], Hanyang Li[1,2,*,+], Lugui Cui[2,+], Shuangqiang Liu[2], Jun Yang[1,*]

[1]Key Lab of In-fiber Integrated Optics, Ministry Education of China, Harbin Engineering University, Harbin 150080, China

[2]Department of Physics, Harbin Engineering University, Harbin 150080, China

[*]hanyang_li@qq.com; yangjun@hrbeu.edu.cn

[+]these authors contributed equally to this work

1. Plasma spectrum collection

To investigate the sources of the driving force that comes from air ionization. A spectrometer was used for plasma spectrum acquisition[1,2]. The corresponding spectrum is shown in Fig. S1(a) (no microsphere in the microstructure) and Fig. S1(b) (microsphere in the microstructure). Several characteristic peaks occur at approximately 560, 616, 643, 703, 773, 833, 878, and 1,062 nm. The characteristic peaks are shown in Table S1.

Table S1. Major characteristic peaks of plasma

Position (nm)	Absorption peak
559	N^+ ion
616, 646, 773, 833	O atom
878	N atom

Images were captured by a high-speed CCD (Figs. S1(I) and S1(II)). The plasma spectrum showed that the laser pulse that was launched from the fibre was beyond the breakdown threshold of air, leading to plasma formation. Figs. S1(a) and S1(b) show that the individual characteristic peaks shifted slightly, which can be ignored in our experiment.

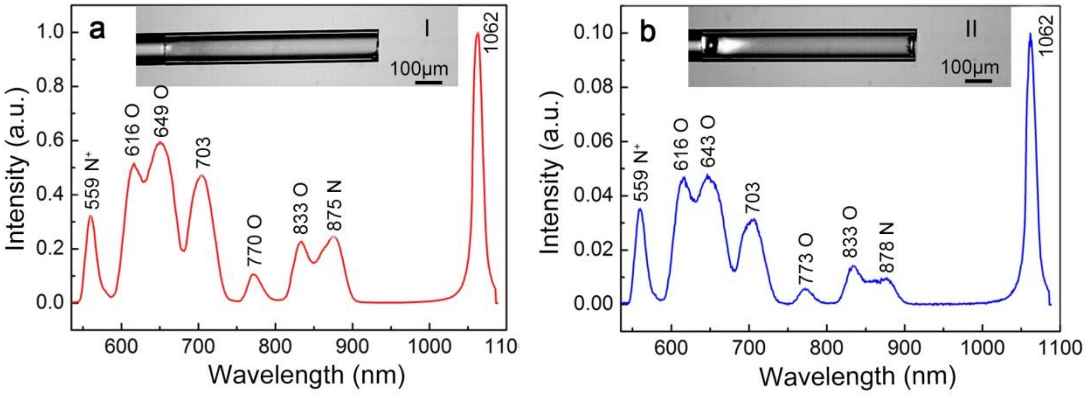

Figure S1. Plasma spectrum. (a) Plasma spectrum for the micro-gun without a microsphere. (b) Plasma spectrum for the micro-gun with a microsphere. (I) and (II) are the images of the micro-gun without and with a microsphere (inset), respectively.

2. Microsphere fragmentation after impact

The maximum distance that the 40 μm microsphere moved was approximately 1,036 μm at the laser pulse energy of 13.74 μJ, and the trajectory approximated a straight line on the glass slide (Fig. S2). We can observe that the microsphere broke down when the nanosecond laser pulse exceeded its damage threshold, as shown in the inset of Figs. S2(a) and S2(b).

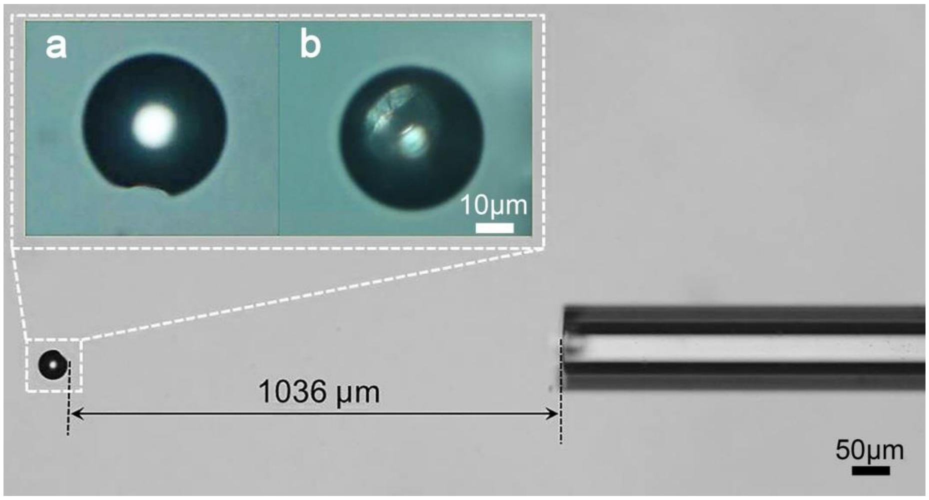

Figure S2. Microsphere with a 40 μm diameter breaks down at the laser pulse energy of 13.74 μJ. (a) Side view. (b) Top view. See Supplementary video 4.

3. Simulation mode setup

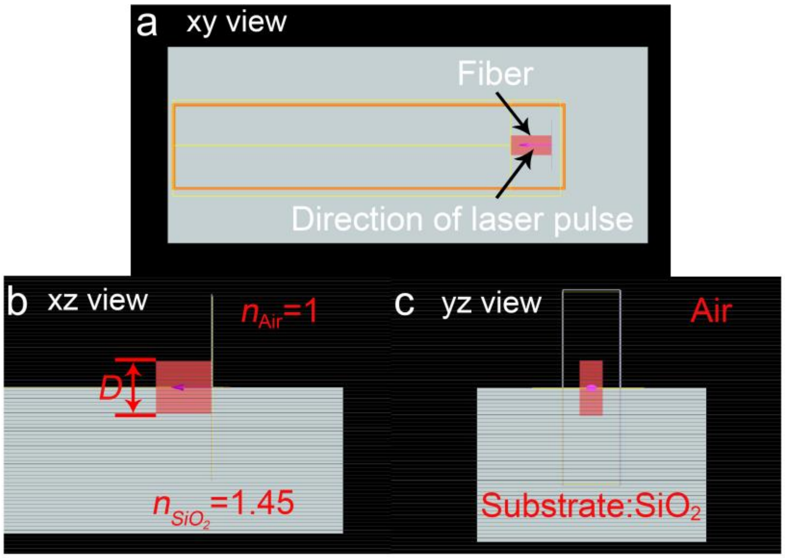

Figure S3. The schematic of the FDTD simulation configuration. (**a**) xy view. (**b**) xz view. (**c**) yz view.

4. Supplementary videos

Supplementary Video 1. Video of the same glass microsphere with a diameter of 50 μm dynamic processes at various laser pulse energies.

Supplementary Video 2. Video of microspheres with diameters from 30 to 60 μm dynamic processes at the same laser pulse energy.

Supplementary Video 3. Video of the microsphere moving with and without a gap.

Supplementary Video 4. Video of the fragmentation of the microsphere after impact.

References

1. Banaee, M. & Tavassoli, S. H. Discrimination of polymers by laser induced breakdown spectroscopy together with the DFA method. Polym. Test. 31, 759-764 (2012).
2. Jiao, L., Truscott, B. S., Liu, H., Ashfold, M. N. R. & Ma, H. H. Imaging spectroscopy of polymer ablation plasmas for laser propulsion applications J. Appl. Phys. 121, 013303 (2017).
